# Teachers’ use of augmented input and responsive strategies in schools for students with intellectual disability: A multiple case study of a communication partner intervention

**DOI:** 10.1177/23969415241290419

**Published:** 2024-10-14

**Authors:** Sofia Wallin, Gunilla Thunberg, Helena Hemmingsson, Jenny Wilder

**Affiliations:** Department of Special Education, Stockholm University, Stockholm, Sweden; 56749 Sahlgrenska University Hospital, Gothenburg, Sweden; 56749 Sahlgrenska University Hospital, Gothenburg, Sweden; Department of Special Education, 123735 Stockholm University, Stockholm, Sweden; Department of Special Education, 123735 Stockholm University, Stockholm, Sweden

**Keywords:** Augmentative and alternative communication, teachers, intervention/therapy, interaction

## Abstract

**Background and aims:**

Teachers serve as critical communication partners for students with intellectual disability (ID) who face communication difficulties. However, teachers may lack sufficient training in using communication partner strategies and augmentative and alternative communication (AAC) in the classroom. This study aimed to explore teacher application of a communication partner intervention (AKKtiv ComPal) in schools for students with ID.

**Methods:**

Video observations were conducted at four schools during a teacher-led group activity at pre- and postintervention, with follow-up 7 months later, focusing on communication partner strategies and AAC use applied as a universal approach in the classroom. Differences and similarities in intervention application and contextual factors that may influence teacher application were investigated using a multiple case study approach, in which the four teachers and their contexts served as the four examined cases.

**Results:**

All cases increased the access to communication boards in the classroom and used more augmented input and responsive strategies following intervention. Follow-up measures revealed variability in augmented input and sustained or more use of responsive strategies. Despite increased strategy use, access to communication boards remained inconsistent, and augmented input was used with variability across observation minutes. Influencing factors to teacher application seemed to be classroom setups (such as having a table), previous AAC skills, student characteristics, and postintervention efforts such as repeating the intervention or participating in follow-up sessions.

**Conclusions:**

This study demonstrates that classroom teachers for students with ID can use augmented input and responsive strategies as a universal design approach in the classroom following the AKKtiv ComPal intervention. However, teachers may utilize the strategies somewhat differently, partly influenced by their contextual factors.

**Implications:**

The findings suggest that while teachers for students with ID can successfully use communication partner strategies in the classroom, their use of augmented input should be nurtured over time to ensure sustained use and possibly improve consistency. Additionally, adapting to the physical environment of the classroom is crucial to optimize the application of these strategies.

## Introduction

Communication is a fundamental right and plays a crucial role in many aspects of life, such as education, social interaction, and achieving personal goals (Brady et al., 2016; Light et al., 2019). However, students with intellectual disability (ID) may face communication difficulties due to speech and language limitations (Sigafoos et al., 2016). Augmentative and alternative communication (AAC) offers tools and strategies to support these students in understanding others and expressing themselves. This includes both unaided modes, such as manual signs, and aided modes, such as photos, AAC symbols, and text, which can be integrated into communication boards or speech-output technologies. Strategies refer to the techniques that AAC users and their communication partners employ to enhance communication and promote language development (Beukelman & Light, 2020).

In educational contexts, teachers serve as critical communication partners for students with ID and communication difficulties (Leatherman & Wegner, 2022). Adequate support in everyday interactions, including access to AAC and models on how to use AAC, can significantly impact student communication skills (Biggs et al., 2018; Chazin et al., 2021). Although teachers acknowledge the importance of AAC, classroom teachers for students with disabilities report challenges in consistent implementation. Such challenges include insufficient AAC education, time constraints, lack of confidence in using AAC, and difficulties in motivating students to use AAC to communicate (Andzik et al., 2017; Biggs & Hacker, 2021; King et al., 2017; Leatherman & Wegner, 2022). Teachers also tend to use AAC more during academic lessons over more spontaneous and social interactions (Andzik et al., 2016; Norburn et al., 2016; Wallin et al., 2023).

Most AAC research focuses on personalized communication systems and factors related to their assessment and implementation (e.g., Biggs et al., 2018; Chazin et al., 2021; Wandin et al., 2023). However, AAC can also be used with a universal approach in the classroom, which involves using AAC tools and strategies designed to be used by school staff and ensure the right to communication and learning for all students, not only those with access to individualized communication systems (Beukelman & Light, 2020; Brady et al., 2016; Tönsing & Dada, 2023). This approach is particularly relevant in school settings, where a significant portion of instruction occurs in group contexts. Furthermore, the selection and access to personalized communication systems often fall outside the control of most school staff (Biggs & Hacker, 2021; Leatherman & Wegner, 2022). Despite this, the universal use of AAC remains relatively unexplored in research.

Augmented input and responsive strategies are two communication partner strategies that can be used with a universal classroom approach and are suggested to facilitate communication and language development in children with communication difficulties (Chazin et al., 2021; McDaniel et al., 2022).

Augmented input involves the partner's use of AAC alongside spoken language during everyday interactions, without expecting a specific response from the person with communication difficulties. It differs from other distinct approaches to partners’ use of AAC, which include prompting specific behaviors or instructing the use of a personalized communication system (e.g., Biggs et al., 2018; Wandin et al., 2023). Augmented input serves as an umbrella term for partners’ use of AAC derived from a sociocultural perspective, encompassing a range of terms with slightly different aims and techniques (e.g., Chazin et al., 2021). In this study, the technique employed is closely aligned with Natural Aided Language, as described by Cafiero (1998). This technique, developed for children with autism spectrum disorders (ASDs), integrates visual symbols as a total immersion strategy into daily activities. Ultimately, students learn these symbols during social interactions in the contexts in which they are used, similar to spoken language development (Cress & Marvin, 2003; Sevcik et al., 2018; Tomasello, 2003). Consequently, augmented input necessitates a varied vocabulary that can be used in a range of communicative functions (Biggs et al., 2018). Numerous reviews suggest that augmented input, especially when combined with other strategies, shows promise in improving communication skills in individuals with communication difficulties (e.g., Biggs et al., 2018; Chazin et al., 2021; Wandin et al., 2023).

Responsive strategies are grounded in theories such as attachment (Ainsworth et al., 2015), sociocultural approaches (Vygotsky, 1980), and parental sensitivity (Warren & Brady, 2007). Fundamentally, these strategies involve using a communication style that is attuned to the child's communication, interpreting, affirming, and expanding on it, without aiming to elicit specific responses (Broberg et al., 2012; Shalev & Hetzroni, 2020). Responsive strategies have been suggested to improve receptive and expressive language, joint attention, and turn-taking in young children, including those with ID and ASD (Girolametto & Weitzman, 2002; McDaniel et al., 2022; Warren & Brady, 2007).

While evidence suggests that augmented input and responsive strategies can facilitate communication and language development in children with ID, there is limited research on how teachers use these strategies following instructional interventions, particularly as a universal classroom approach. Understanding teacher behavior is vital, as student outcomes depend on how teachers apply such interventions. Previous research on augmented input has largely focused on researcher-delivered interventions (Chazin et al., 2021) and responsive strategies on parents (Shalev & Hetzroni, 2020). Although limited research has examined the use of communication partner strategies by school staff following interventions, the studies that exist have shown promising application and outcomes. However, they have focused primarily on preschools or schools for students with physical disabilities without ID and their personalized communication systems (Chang et al., 2016; Lawton & Kasari, 2012; McConachie & Pennington, 1997; Popich & Alant, 1999; Senner & Baud, 2017).

This study builds on existing research by exploring a communication partner intervention, AKKtiv ComPal, in real-world school settings for students with ID. The intervention is designed to promote a communicative classroom environment where school staff employ a universal approach to support communication development among all students. AKKtiv ComPal is part of the AKKtiv program (AKKtiv, 2024), which is rooted in child language development theories that emphasize the role of adults as facilitators (Bruner, 1974; Vygotsky, 1980). The program offers various intervention packages for different partner groups, all of which include educational sessions on communication and AAC, both theoretical and practical. Research focusing on the AKKtiv program has mainly focused on parents in AKKtiv ComAlong and AKKtiv ComAlong Toddler, showing that parents report increased use of augmented input and responsive strategies following intervention (Fäldt et al., 2020; Ferm et al., 2011; Jonsson et al., 2011; Rensfeldt Flink et al., 2022). In observational studies, Rensfeldt Flink et al. (2022) observed no behavior changes in parents of children with severe and profound intellectual and multiple disabilities, while others (Broberg et al., 2012; Jonsson et al., 2011) noted increased strategy use in parents of a wider range of children postintervention.

This article is the first to focus on the AKKtiv ComPal intervention, the only AKKtiv package specifically targeting school staff. The study incorporates feasibility aspects by examining how teachers utilize the intervention as intended and identifying factors that may influence its application. The research questions posed were: How do teachers use augmented input and responsive strategies as a universal approach in their classrooms following the AKKtiv ComPal intervention? What differences and similarities in strategy use can be identified across teachers, and why might such differences and similarities occur?

## Method

### Research design

To examine a communication partner intervention (AKKtiv ComPal) as employed by four teachers in classrooms for students with ID, pre-, post-, and follow-up data were collected using video observations during a teacher-led group activity. A multiple case study design was used, as it is considered suitable for studying complex interventions within their natural context, providing a real-world perspective on the application of the intervention (Creswell, 2022; Yin, 2018). Each teacher and their respective school context served as individual cases. A within-case analysis was conducted to assess how each teacher applied augmented input, including access to communication boards, and responsive strategies used as a universal approach in their classrooms. Subsequently, a cross-case analysis was conducted to examine differences and similarities across cases, allowing for the identification of potential patterns in strategy application across cases.

### Participants

Four schools with classrooms for students with ID, located close to one of the largest municipalities in Sweden, participated in the study. As part of a larger study, Wallin et al. 2023 described the recruitment process in detail. One teacher from each of the four schools participated. Classroom and teacher characteristics are summarized in Table 1. All participants worked as classroom teachers with students who followed the curriculum for students with severe ID, which focuses on five subject areas: physical coordination, aesthetics, everyday activities, perception of reality, and communication. In each of these classrooms, most students also had ASD. One student in classroom A had a personalized AAC system (a book based on communication boards) provided by speech-language pathologists at the habilitation center, but this was not being used in school at the beginning of this study.

**Table 1.** Teacher and student characteristics.

Characteristics	Case A	Case B	Case C	Case D
Classroom				
Total number of students	3	3	4	5
Setting	Self-contained	Self-contained	Special school	Self-contained
Curriculum	Severe ID	Severe ID	Severe ID	Severe ID
Teachers				
Pseudonym	Anna	Beatrice	Christina	Diana
Age	39	38	61	55
Sex	Female	Female	Female	Female
University degree	Teacher with ID specialization	Teacher	Teacher with ID specialization	Teacher
Years of experience (schools for students with ID)	6	5	4	0.25^a^
AAC education	Several	Manual signs	Manual signs	None
AAC experience	Yes	Yes	Yes	Yes
Students				
Age range	6–8	6–8	7–11	6–9
Additional diagnoses	ASD, genetic disorders, epilepsy	ASD, genetic disorders, ADHD	ASD, epilepsy	ASD
Student communication	Vocalizations, pictures, manual signs, body language	Speech, pictures, manual signs, speech-generating device	A few words, pictures, manual signs, pointing	Pictures, manual signs, pointing
Had personalized communication system	One student: a book based on communication boards	None	None	None

*Note.* AAC = augmentative and alternative communication; ID = intellectual disability; ASD = autism spectrum disorders; ADHD = attention deficit hyperactivity disorder. Teacher information was self-reported; student information was reported by parents.

^a^ Had experience working with the target group in other settings.

Throughout this article, these teachers will be identified using the pseudonyms Anna (Case A), Beatrice (Case B), Christina (Case C), and Diana (Case D).

### Intervention content and procedures

The AKKtiv ComPal intervention consisted of three parts: a preparatory instructor education, the intervention, and an instructor follow-up session.

The 2-day preparatory instructor education prepared ComPal instructors to lead the intervention at their schools. They were provided with ready-made PowerPoint presentations for the intervention sessions and 21 ready-made communication board templates in multiple software formats. The boards, each containing 24 AAC symbols, were organized similar to those described by Jonsson et al. (2011).

**Figure 1.**
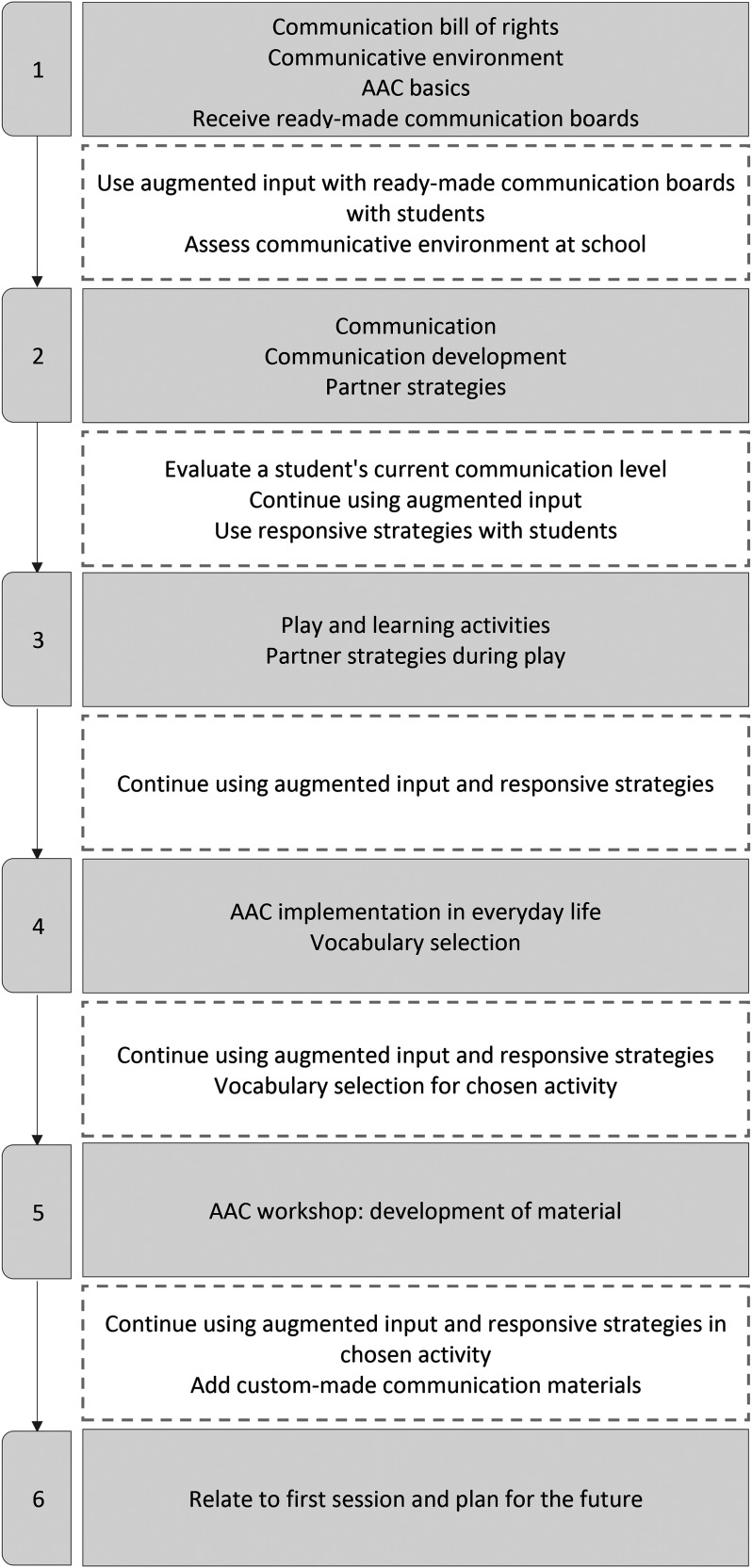
Intervention content. Gray fields indicate sessions, white fields indicate assignments.

*Note.* Communicative environment includes AAC as a universal design approach in the classroom and AAC use throughout the day. AAC = augmentative and alternative communication.

The intervention involved all school staff in the studied classrooms across six educational sessions totaling 14 hr, with additional in-between-session assignments (see Figure 1 for details). Augmented input was introduced to the participants as a language immersion strategy called point-talking, where spoken language is augmented by showing or pointing at objects, people, or pictures, as often as possible. The intervention emphasized the use of augmented input across various communication functions, mirroring the use of natural spoken language. Guidance was provided in vocabulary selection for custom-made boards, emphasizing both core vocabulary (e.g., yes, no, help, fun), and fringe vocabulary specific to particular contexts or activities. Responsive strategies were presented using a “three-stage rocket” model: (a) Observe and Listen, (b) Wait and Expect, and (c) Interpret and Respond, to stimulate natural and enjoyable interactions.

The instructor follow-up session was a full-day session carried out 6 months postintervention. Its main objective was to share experiences and collectively discuss the challenges and facilitators encountered while leading the intervention.

Anna, Beatrice, and Christina, chosen by their principals for their AAC knowledge and willingness to coach colleagues, participated in both the preparatory instructor education and follow-up session, held by an AAC specialist at a Swedish healthcare communication center. Diana, newly employed without AAC education, was not selected as an instructor. Consequently, Anna, Beatrice, and Christina held all intervention sessions at their schools. Anna held the intervention twice. Diana participated in five sessions led by a special needs coordinator who had completed the preparatory instructor education. The intervention was carried out independently by the ComPal instructors without involvement from AAC specialists. The authors were not involved in any step of the intervention.

### Data collection

To study the teachers’ application of the intervention, video observations were conducted during circle time, a routine-based teacher-led group activity focusing on academics and socialization, such as attendance, schedule, and weather, typically lasting 15–30 min (Wallin et al., 2023). The four teachers conducted the activity as usual. They were aware of being video recorded but did not know what specific aspects would be studied, other than that it was related to the intervention. To capture multiple angles, account for repositioning, and not interfere in the classroom, five wall-mounted iPads were used. These iPad screens were duplicated on a computer running Windows 10 using Reflector, version 3.2.0 (Squirrels, LLC, 2018) enabling the researcher to monitor the cameras remotely.

**Figure 2.**
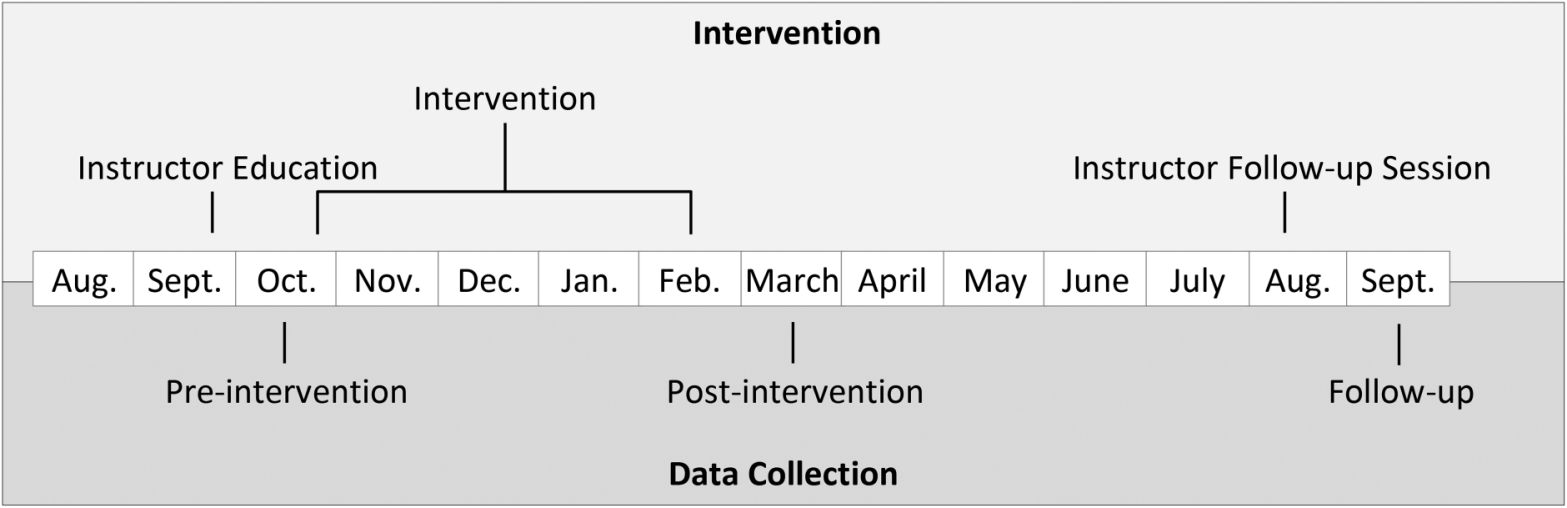
Intervention and data collection timeline (year 2019–2020).

Observations were conducted once in each classroom at pre- and postintervention, that is, in October 2019 and February 2020, with follow-up observations 7 months later, in September 2020. In total, three observations in each classroom. See the study timeline in Figure 2.

### Analysis

In relation to the intervention, it was expected that teachers would make communication boards available in the classroom and use them for augmented input, as well as using responsive strategies. Consequently, these three components were selected for assessing the application of the intervention based on the video observations collected at pre- and postintervention as well as at follow-up video observation after the instructor follow-up session (see Figure 2).

#### Communication boards

A communication board was defined as pictures organized in rows and columns in a grid pattern, either on paper or in speech-output technologies. It had to contain core vocabulary to be registered as a communication board, which excluded, for example, schedule systems. The communication boards were counted once per page. Personalized communication systems based on communication boards were counted once per whole system.

Additionally, notes were taken regarding the type of symbols used (e.g., photos, AAC symbols), grid sizes, overall vocabulary, location in the classroom, and whether they were ready-made (provided through the intervention) or custom-made.

#### Augmented input

Although the intervention includes pointing to people and objects in the use of augmented input (point-talking), this study specifically focuses on the use of pictures, both with and without speech-output, as is commonly utilized in earlier research (e.g., Biggs et al., 2018; Chazin et al., 2021; Wandin et al., 2023).

Frequency of augmented input was measured by counting each time the teacher showed or pointed at a picture alongside a spoken word/utterance (using their own voice) related to that picture. Repeated showings or pointings were counted only if the corresponding speech was also repeated, and vice versa. When the spoken utterance did not include the symbolized word or phrase, as when students were asked to name a picture, it was not counted.

Additionally, notes were taken regarding materials (e.g., communication boards, single pictures, speech-output technologies) and the topics used in augmented input.

#### Responsive strategies

Responsive strategies included aspects described in the intervention as the three-stage rocket (observe and listen, wait and expect, interpret and respond), along with aspects aiming to promote communication (i.e., being physically close to the students, following students’ focus of interest, and using AAC).

Teacher use of responsive strategies was measured using the Responsive Augmentative and Alternative Communication Style (RAACS) scale version 4 (Lindberger et al., 2024). This scale was selected for its inclusion of AAC as a responsive strategy, its psychometric properties, and its previous use in studies related to the AKKtiv program (e.g., Broberg et al., 2012; Rensfeldt Flink et al., 2022). Version 3 of the scale has shown fair-to-perfect interrater reliability and excellent internal consistency (Broberg et al., 2012; Wandin et al., 2022). In this study, version 4 was used because of its higher interrater reliability and reduced incidence of ceiling effects compared to version 3 (Lindberger et al., 2024). While originally designed for parent–child interactions, the RAACS scale has also been used to examine the responsive strategies of a trained therapist and was suggested not to be specific to parenting (Wandin et al., 2022). In the present study, the terminology of the RAACS scale was adapted to replace “parent” with “teacher” and “child” with “students,” but no other modifications were made. Version 4 of the scale comprises seven items scored minute-by-minute and three scored globally. The scale generates a RAACS score ranging from 3 to 23, with a higher score indicating a higher use of responsive strategies. See an overview of the instrument and its items in Table 2. For comprehensive details on the instrument, the reader is referred to Lindberger et al. (2024).

**Table 2.** Items of the Responsive Augmentative and Alternative Communication Style (RAACS) scale, used to measure responsive strategies.

Items
Minute-by-minute items (Scale 0–2)
1. The teacher is attentive to and confirms the students’ communication (e.g., Repeating students’ utterances. Labeling students’ physical and communicative actions. Commenting.)
2. The teacher adjusts physically to the students (e.g., The teacher is being turned to the students or positioned to enable communication according to the activity.)
3. The teacher gives the students space to communicate (e.g., Observing and waiting expectantly. Being expectant and encouraging even if students do not respond.)
4. The teacher clarifies their own communication (e.g., Emphasizing important words. Using varied intonation, short utterances, and uncomplicated language.)
5. The teacher communicates according to the students’ focus of interest or conversational topic (e.g., Following in the play/activity. Commenting, telling, and explaining. Imitating the students’ actions.)
6. The teacher expands on the students’ communication (e.g., Putting the students’ actions into words. Repeating and developing the content of students’ communication.)
7. The teacher uses AAC (e.g., Using available AAC. Using signs together with speech. Using signs and symbols in songs and chants.)
Global items (Scale 1–3)
8. The teacher is engaged
9. The teacher adapts to the student
10. The teacher adjusts to the communicative level of the student
RAACS score
Calculated by using the mean of each of the seven minute-by-minute scores added to the scores of the three global items. A higher score means more use of responsive strategies and range between 3 and 23.

*Note.* Low item scores indicate low use of the strategy, higher scores indicate consistent and frequent use of the strategy.

#### Procedures

The first 10 min of each video observation were used in the analysis, totally comprising 120 min to analyze. Using 10-min sections was determined according to the RAACS coding manual, and the first minutes were selected based on having the most similar content across time points. The mean duration of the uncut videos was 14 min 3 s (*SD *= 2 min 56 s) preintervention, 16 min 4 s (*SD *= 3 min 22 s) postintervention, and 16 min 38 s (*SD *= 2 min 25 s) at follow-up. The same sections were used to analyze all measures. Communication boards were counted based on availability, globally within the 10-min section. Augmented input was counted minute-by-minute, and RAACS used a combination; see Table 2.

The multiple case analysis was conducted in two steps, following Creswell's (2022) and Yin's (2018) recommendations for conducting a cross-case analysis. First, each case was independently analyzed (within-case analysis) to build an understanding of how each teacher used the partner strategies. Findings were presented using summarized descriptions, data visualizations, and descriptive statistics. Quotations of utterances were also used, with the specific words used with augmented input highlighted by underlining and capitalization. Second, within-case findings were synthesized and compared to identify similarities and differences across cases (cross-case analysis). This synthesis included: (a) application of communication partner strategies over time, (b) mean deviation from the group mean, calculated by adding the deviation of each case from the group mean at three observation time points and then dividing by three, and (c) other prominent aspects that emerged during the within-case analysis. Finally, contextual similarities and differences (such as staff and student characteristics, classroom setups, and intervention roles) were included into the cross-case analysis to potentially add insights into factors that may influence the intervention application.

### Reliability

To assess interrater reliability (inter-RR) of augmented input frequency and the RAACS scale, a second rater, blinded to the study stage, analyzed 20% of the video data. Both raters were trained on videos of circle time activity from other schools, following an iterative process of independent coding, comparison, and discussion until consensus was reached on the coding of augmented input and interpretations of the RAACS items. For intrarater reliability (intra-RR), the first rater reanalyzed 20% of the study data.

The intraclass correlation coefficient for augmented input was .94 for inter-RR and .97 for intra-RR, both indicating excellent reliability (Koo & Li, 2016).

All items of the RAACS scale were converted to the same three-point scale before statistical analysis. Cohen's kappa for inter-RR was *κ *= .62, and *κ *= .70 for intra-RR. According to the interpretative framework proposed by Landis and Koch (1977), which is widely used in behavioral studies, both values indicate substantial agreement.

### Ethical considerations

Ethical approval was obtained from the Swedish Ethical Review Authority (Reference No. 2019-03994). The four teachers consented to participate in the study, and the classroom attendees consented to be seen on the video observations. When someone declined to be video recorded, they were relocated to a separate room during data collection. Staff members were instructed to be attentive to any signs of discomfort expressed by the students during video observations, which would immediately be terminated. This situation did not arise. The voluntariness of school staff to become ComPal instructors may have been influenced by their selection by principals. However, partaking in the ComPal instructor education did not obligate involvement in the research study. In fact, two schools decided not to participate after this education.

## Results

### Within-case findings

**Figure 3.**
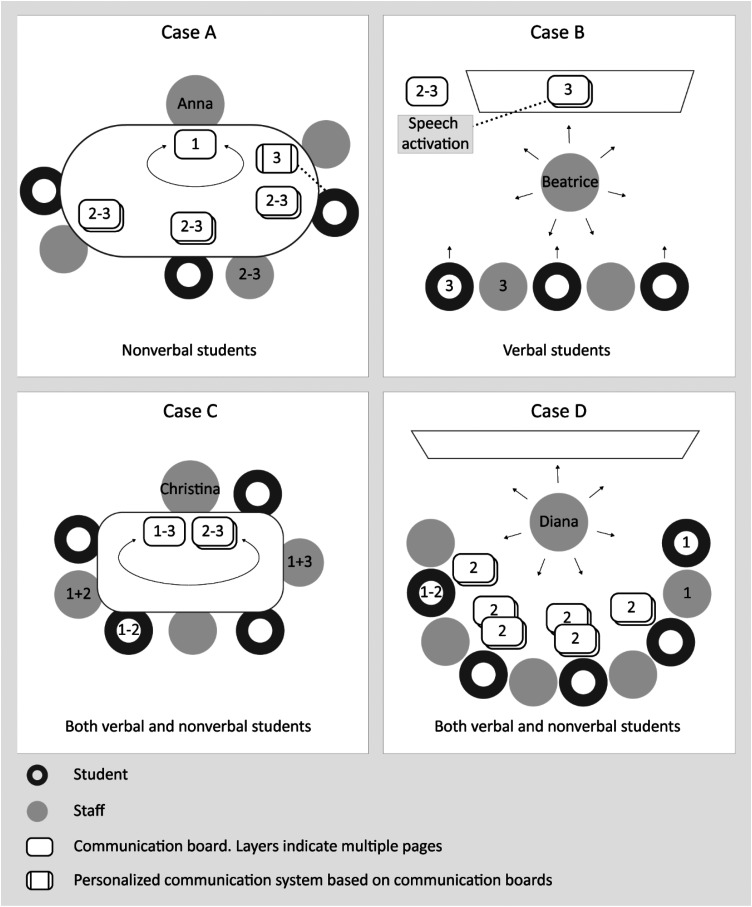
Classroom setup and position of communication boards (top view).

*Note.* Numbers represent the observation in which they were present. No number means present in all observations. Students were defined as verbal/nonverbal based on the examined video observations.

**Figure 4.**
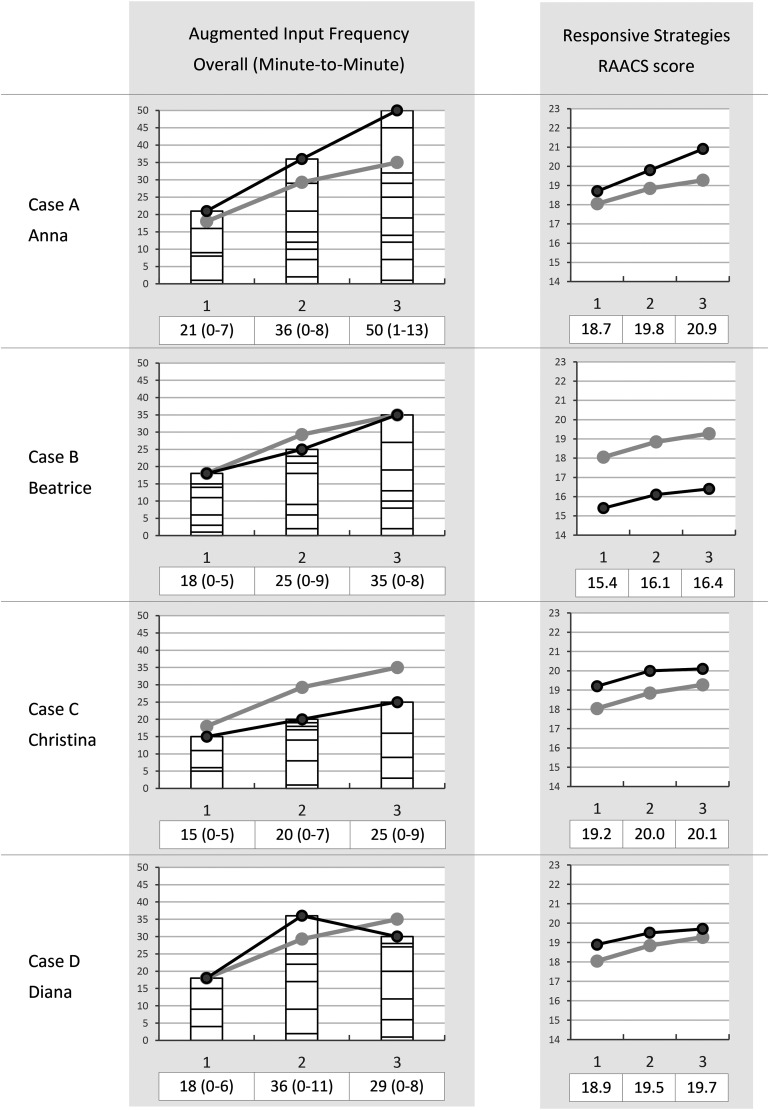
Each teacher's use of augmented input (frequency) and responsive strategies (RAACS score) at three time points. Gray lines represent the mean of all four teachers.

*Note.* RAACS = Responsive Augmentative and Alternative Communication Style scale.

The within-case findings will be presented case-by-case, comprising descriptions of the classroom setup during the circle time activity, including the access of AAC tools, and the use of augmented input and responsive strategies. Classroom setups and placements of communication boards are visualized in Figure 3. Individual data on the overall use of augmented input (frequency) and responsive strategies (RAACS score) at the three time points are visualized in Figure 4.

#### Case A: Anna

In the circle time activity in Anna's classroom, three students, two to three school staff members, and two personal assistants participated during the three observations (staff/student ratio: *M *= 1.22). All subactivities were performed seated around a large oval table. None of the students used verbal communication interpreted as words during any observation.

At pre-intervention, one communication board in a 2 × 6 grid pattern with AAC symbols with corresponding text underneath was available. It included weekday colors and an “I don't know” option, used during the day-of-the-week subactivity, with Anna moving it toward students in a given order. Throughout the post- and follow-up observations, each of the three students had a multiple-page communication board placed at their seats. The number of pages increased from four postintervention to eight at follow-up (i.e., a total of 12 and 24 boards, respectively). These boards were custom-made and contained 3 × 4 grids with AAC symbols and photos, with corresponding text underneath. They included vocabulary items such as circle time, begin, end, good morning, names of people, here, not here, soon, yes, no, and weather. Furthermore, one student had access to a personalized multipage communication system at follow-up, which they had not had access to in previous observations. Additional aided AAC included single pictures (people, the day's name, and weather), a single-switch speech-output technology for announcing presence, and a speech-output schedule.

Anna's frequency of augmented input increased from an initial frequency of 21 to 36 postintervention and 50 at follow-up. She used augmented input on communication boards, the speech-output schedule, and single pictures. In all observations, she used augmented input when talking about the calendar, weather, attendance, and today's schedule, for example “FRIDAY is YELLOW” and “I see it's the BROWN day we call THURSDAY,” and some more general words: “Do you think it SMELLS GOOD? Or BAD? YUCK.” She mostly used augmented input with one word per sentence, but over time she used augmented input with up to three words per sentence. She had a high variability in frequency of augmented input throughout the 10 min of observation at all time points. At pre- and postintervention, 5 and 8 min comprised augmented input, respectively. At follow-up, she used augmented input to some amount in all 10 min but to a great range.

In all three observations, Anna consistently employed responsive strategies, starting with a RAACS score of 18.7 out of a maximum of 23. Her score increased with every observation, to 19.8 postintervention and 20.9 at follow-up. Over time, she increasingly gave students more space to communicate, resulting in a distinct score change over time on Item 3 (preintervention, *M *= 1.10; postintervention, *M *= 1.50; follow-up, *M *= 1.60). She also communicated according to the child's focus of interest or conversational topic to a gradually higher degree, leading to a distinct score change on Item 5 (preintervention, *M *= 0.90; postintervention, *M *= 1.10; follow-up, *M *= 1.40). Finally, she gradually expanded more on students’ communication, which led to higher scores over time on item 6 (preintervention, *M *= 0.30; postintervention, *M *= 0.70; follow-up, *M *= 1.20). Across observations, she could easily turn to and reach to students from her position at the table. She also consistently clarified her communication and used various AAC tools, including those previously described and manual signs. For these reasons, she had ceiling effects on Items 2, 4, and 7 (see Table 2).

#### Case B: Beatrice

During the circle time activity in Beatrice's classroom, two to three students and two to three staff participated (staff/student ratio: *M *= 0.88). Beatrice was seated on a rolling teacher stool close to a wall-mounted whiteboard, while the other attendees were in a row facing her. Subactivities were presented on the whiteboard or at the students’ seats. All students used some spoken language during the observations.

No communication board was present during the first observation in Beatrice's classroom. Postintervention and at follow-up, a custom-made communication board was mounted on the wall next to the whiteboard. It contained core vocabulary items such as happy, funny, silly, wrong, and again, using AAC symbols and corresponding text underneath. Postintervention, the grid pattern was 2 × 3, increasing to 2 × 6 at follow-up. At follow-up, an iPad communication app with speech-output was projected on the whiteboard during sub-activities related to the days of the week and weather and season, with an “it's” expression on all pages. A total of four pages with various grid sizes, ranging from 3 × 3 to 5 × 7, with AAC symbols and corresponding text underneath were projected. Each page also had a message field at the top. The speech-output was activated from the iPad, which was placed on a table next to the whiteboard. Additional aided AAC tools comprised single pictures (people, weather, and emotions), a list of numbers, and a schedule system. All AAC tools were mounted on the whiteboard, and students approached the whiteboard in a given order as provided by Beatrice.

Beatrice's frequency of augmented input within the ten minutes of observation increased from 18 preintervention to 25 postintervention and 35 at follow-up. She used augmented input on communication boards (both paper-based and on the iPad), and single pictures. She consistently used augmented input when talking about the calendar, attendance, today's feelings, weather, and today's schedule, such as “So you’re AWAKE and HAPPY,” and “TODAY it's TUESDAY, 15^TH^.” She mostly used augmented input on one word per sentence, but occasionally on up to three words in a sentence at follow-up. At postintervention and follow-up, she also used more general expressions such as in “I did it WRONG, haha. I must try AGAIN.” She used augmented input to some amount in seven of the 10 min in all three observations, with a high variability in frequency across minutes.

Throughout the three observations, Beatrice used a relatively high level of responsive strategies. Her RAACS score increased from an initial 15.4 out of a maximum of 23 preintervention to 16.1 postintervention and 16.4 at follow-up. Over time, she expanded more on students’ communication, leading to higher scores on Item 6 (preintervention, *M *= 0.70; postintervention, *M *= 0.80; follow-up, *M *= 1.20). Across observations, she clarified her communication and used various AAC, including those previously described and frequent use of manual signs, resulting in ceiling effects on Items 4 and 7 (see Table 2).

#### Case C: Christina

The circle time activity in Christina's classroom involved three to four students and three to four staff (staff/student ratio: *M *= 0.91). All subactivities were performed seated around a table. Two students did not use verbal communication interpreted as words, while one used single spoken words. An additional student who used spoken sentences participated in the first two observations.

No communication board was accessible at preintervention. At postintervention and follow-up, a custom-made double-paged communication board was present. It had a 5 × 5 grid of AAC symbols with corresponding text underneath, containing vocabulary specific to the activity, such as weather, and calendar, as well as more general expressions such as happy, don’t know, and how many. This board was available throughout the postintervention observation and was partially available at follow-up. Additional aided AAC included a speech-output technology used to select songs and single pictures (the day's name, weather, and the activities of the day). Preintervention, they also had access to a page with text sentences with the phrase “How many children/students are here today,” with pictorial support for each word. This text was later incorporated into the vocabulary of the communication board.

Christina's frequency of augmented input increased from pre- to postintervention, from an initial total of 15 to 20 and finally 25 at follow-up. She used augmented input on communication boards and single pictures. She also used the speech-output technology to select songs a few times, but since she did not use her own voice to augment the output, this was not counted as augmented input. In all observations, she used augmented input when talking about the calendar, weather, attendance, and today's schedule, such as “The SUN will shine soon.” At postintervention she also used augmented input with the more general word happy: “It makes me so HAPPY when you are happy.” She consistently used augmented input with one word per sentence. Throughout all observations, students reached for the communication board and pictures and often grabbed them, interrupting Christina's intended use of augmented input multiple times. She used augmented input with high variability in frequency across the 10 min of observation at all time points. Initially, she used some augmented input in four of the observed minutes, then seven at postintervention, and again in four of the minutes at follow-up.

In all three observations, Christina used responsive strategies to a high degree. Her RAACS score increased from 19.2 out of a maximum of 23 to 20 postintervention. At follow-up, her score was stable (20.1). Over time, Christina became more attentive and confirming to students’ communication, leading to a distinct score change on Item 1 (preintervention, *M *= 1.30; postintervention, *M *= 1.50; follow-up, *M *= 1.80). Across observations, she could easily turn to and reach students from her position at the table. She also consistently clarified her communication and used various AAC tools, including those previously described, and manual signs. For these reasons, she had ceiling effects on Items 2, 4, and 7 (see Table 2).

#### Case D: Diana

In the circle time activity in Diana's classroom, three to five students and five to six staff participated (staff/student ratio: *M *= 1.23). Diana was seated on a rolling teacher stool close to a wall-mounted whiteboard, with the other attendees seated in a half-circle facing her. Subactivities were presented on the whiteboard or at the students’ seats. Three students did not use verbal communication interpreted as words, while two used single spoken words.

No communication board was present at preintervention. At postintervention, each of the four students received a double-paged custom-made communication board each, containing vocabulary related to the calendar and weather, as well as yes, no, and I don’t know, organized in a 3 × 3 and 2 × 4 grid pattern, respectively. Two students had additional access to a double-paged communication board with numbers and months (grid sizes 6 × 6 and 4 × 3). In total, 12 boards were present, all containing AAC-symbols and pictures representing how to manually sign the words and corresponding text underneath. The boards were only accessible during parts of the circle time activity, when going through the calendar and weather. At follow-up, no communication board was accessible. Additional accessible AAC was single pictures (weather, people, and schedule) and physical objects representing songs.

Diana's use of augmented input increased from an initial frequency of 18 to 36 postintervention. At follow-up, the frequency decreased to 30. She used augmented input with single pictures and communication boards. She consistently used augmented input when talking about the calendar, weather, and today's schedule, such as “GYM CLASS is on THURSDAY” and “Today, it's TUESDAY.” She mostly used augmented input on one word per sentence, but occasionally on 2 times per sentence at postintervention. She used augmented input with high variability in frequency across the ten minutes of observation at all time points. The number of minutes in which she used augmented input to some amount was initially four, then six at postintervention, and then seven at follow-up.

Throughout the three observations, Diana used a high degree of responsive strategies. Her RAACS score increased from 18.9 out of a maximum of 23 to 19.5 postintervention and 19.7 at follow-up. However, she did not display any distinct score changes over time on any of the RAACS items. She consistently clarified her communication and used various AAC, including those previously described and manual signs, resulting in ceiling effects on Items 4 and 7 (see Table 2).

### Cross-case findings

The cross-case findings will be presented in two sections, one highlighting similarities across cases and the other focusing on differences. The cross-case synthesis is shown in Table 3.

**Table 3.** Cross-case synthesis.

Aspect	Case A	Case B	Case C	Case D
Communication boards				
Number				
Preintervention	1	0	0	0
Comparison pre–post	+11	+1	+2	+12
Comparison post–follow-up	+13	+4	±0	−12
Mean deviation from group mean	+7.67	−3.00	−3.67	−1.00
Custom/ready-made boards	Custom	Custom	Custom	Custom
Augmented input				
Frequency				
Preintervention	21	18	15	18
Comparison pre–post	+15	+7	+5	+18
Comparison post–follow-up	+14	+10	+5	−6
Mean deviation from group mean	+8.25	−1.42	−7.42	+0.58
Variability across minutes	High	High	High	High
Responsive strategies				
RAACS score				
Preintervention	18.7	15.4	19.2	18.9
Comparison pre–post	+1.1	+0.7	+0.8	+0.6
Comparison post–follow-up	+1.1	+0.3	+0.1	+0.2
Mean deviation from group mean	+1.08	−2.76	+1.04	+0.64
RAACS items				
Ceiling effects	2, 4, 7	4, 7	2, 4, 7	4, 7
Distinct score changes over time	3, 5, 6	6	1	None

*Note.* Mean deviation from the group mean: The mean value of each case's deviation from the group means in all three observation time points.

#### Similarities across cases

Four similarities in teacher application were found across cases. First, all four cases displayed higher scores in all partner strategies at postintervention (number of communication boards, augmented input frequency, and responsive strategies). Second, there was a high minute-by-minute variability in the use of augmented input across data points. Third, teachers opted to use custom-made communication boards rather than the provided ready-made ones. Fourth, ceiling effects were noted on the RAACS scale related to clarifying their own communication and using AAC (Items 4 and 7). Regarding contextual factors, a shared characteristic among all classrooms was the consistently high staff/student ratio.

#### Differences across cases

Several differences were found across cases. Each case stood out from the other ones in at least three aspects.

Case A, Anna, used a higher frequency of augmented input, with a mean deviation from the group mean of 8.25. She was also the only case who had distinct score changes in three minute-by-minute items of RAACS (the other displayed such change in maximum one item). Additionally, this was also the only case with higher scores in all strategies at post- and follow-up observations. Contextual factors that differed from the other cases were that this teacher had the most previous AAC education, and all students in this classroom were nonverbal, contrary to the other cases. Additionally, this teacher conducted the intervention twice, whereas the other cases only conducted or participated in it once.

Case B, Beatrice, had generally lower scores on responsive strategies than the other cases, with a mean deviation from the group mean of −2.76. In this case, contrary to the other cases, all AAC tools were mounted on the whiteboard and the teacher decided who and when to use them. A contextual factor that differed from the other cases was that all students in this classroom used spoken language.

Case C, Christina, had a generally lower augmented input frequency than the other cases, with a mean deviation from the group mean of −7.42. Unlike the other cases, this teacher's use of augmented input was occasionally interrupted by students grabbing the communication boards or pictures. This case was also the only case who displayed a distinct score change on Item 1 in RAACS, related to being attentive and confirming to students’ communication.

Case D, Diana, was the only case that displayed lower number of communication boards and frequency of augmented input at follow-up observations compared to postintervention. Furthermore, unlike the other cases, she did not show any distinct score changes over time in any of the RAACS items. Contextual factors that differed from the other cases were that this teacher did not participate in the instructor education or the instructor follow-up session, as she was not an instructor. This teacher also had the least experience working in schools for students with ID and had no previous AAC education.

A factor that differed across all cases was the variation in the number of communication boards. In cases A and D, each student received their own set of boards, as opposed to B and C where all students and staff shared the same boards.

## Discussion

This study examined how teachers in classrooms for students with ID used augmented input (including access to communication boards) and responsive strategies as a universal approach in the classroom following the AKKtiv ComPal intervention. Based on video observations, the results provide descriptions of each teacher’s strategy application in the classroom and similarities and differences across teachers.

The following discussion will be structured around the four identified similarities in application across cases, with the following sections: (a) application over time, (b) minute-by-minute variability, and (c) constant factors, including the use of custom-made communication boards and the ceiling effects of RAACS. Each section will also address differences observed across cases and explore possible reasons why these application similarities and differences might have emerged.

### Application over time

Results revealed increased access to communication boards, higher frequency of augmented input, and a higher degree of responsive strategies postintervention across all cases, reflecting the findings of earlier research in other settings (i.e., Chang et al., 2016; Lawton & Kasari, 2012; McConachie & Pennington, 1997; Popich & Alant, 1999; Senner & Baud, 2017). These findings are encouraging, as partner strategies have been shown to positively impact communication and language development in children with communication difficulties (Chazin et al., 2021; McDaniel et al., 2022). However, their strategy application also differed across cases, especially at follow-up.

The frequency of augmented input appeared to correlate with the access to communication boards, as all cases had similar score changes in both aspects in each data collection (see the specific scores in Table 3). Case A, with most communication board access, used augmented input most consistently and frequently, while Case D, with no access at follow-up, decreased in augmented input frequency. Despite this correlation, case D's frequency of augmented input did not decrease as dramatically as the number of communication boards, meaning she continued to use augmented input with single pictures, such as those representing different weather. While this indicates improved augmented input skills, it also highlights a potential risk of limiting students’ exposure to aided AAC use across multiple communicative functions. Given the assumption that students learn symbols through social interactions (Cress & Marvin, 2003; Sevcik et al., 2018; Tomasello, 2003), ensuring access to a varied vocabulary for augmented input is crucial (Biggs et al., 2018), which is a key aspect of the AKKtiv ComPal intervention. Case D's lower scores at follow-up could be linked to not participating in the follow-up session (as this teacher was not an instructor). This observation aligns with findings by Senner and Baud (2017), who highlighted the need for postintervention support in their study examining school staff in a classroom where students used speech-output technologies.

Responsive strategies were most consistently used over time across cases, with higher scores postintervention and stable (Cases B-D) or higher scores (Case A) at follow-up. This aligns with existing research on teachers implementing responsive strategies, which has consistently shown improved skills postintervention (Chang et al., 2016; Lawton & Kasari, 2012; McConachie & Pennington, 1997; Popich & Alant, 1999). Studies with follow-up measures also demonstrate stability or continued improvement (Chang et al., 2016; McConachie & Pennington, 1997). Interestingly, it appears that the teachers in this study applied responsive strategies in somewhat varied ways, as no clear patterns across teachers emerged in any of the 10 items of RAACS.

Case A stood out among the other cases by displaying higher scores in all strategies at follow-up observations compared to postintervention, possibly because this teacher conducted the intervention twice. This finding indicates that postintervention support, as suggested earlier, may not necessitate external support, as this teacher improved her skills simply by repeating the intervention. Earlier research has also suggested that staff with more AAC training and experience more easily utilize communication partner strategies (Douglas, 2012; McConachie & Pennington, 1997). In this study, this aspect would not only explain the higher use of strategies in Case A, but also the decreasing use of augmented input in Case D, of which the teacher had the least AAC education and experience working in schools for students with ID.

### Minute-by-minute variability in augmented input

The findings revealed that augmented input frequency varied minute-by-minute across cases and time points, partly due to inconsistent access to communication boards. Only Cases A and C had continuous board access, most likely because they could place them on their tables. All cases consistently used communication boards in routine-based subactivities for academic and informational purposes, such as attendance, days of the week, and weather, likely influencing the minute-by-minute variability. Previous research has shown that teachers tend to focus their AAC use on academic purposes (Norburn et al., 2016), and Beukelman and Light (2020) propose that it is easier to become familiar with the vocabulary and have access to aided AAC in activities with similar content from day-to-day. While this approach helps teachers become familiar with augmented input, it is vital to integrate its use into more spontaneous interactions. Considering that the teachers were relatively new to augmented input before the intervention, providing additional support as they become more comfortable using it in routine-based interactions might further facilitate this integration.

### Constant factors

All cases used custom-made communication boards instead of the provided ready-made ones. This finding underscores the importance of having software tools available for developing communication boards. For this reason, vocabulary selection, with an emphasis on using a varied vocabulary as suggested by Biggs et al. (2018), seems to be an important component of the intervention curriculum. The cases also demonstrated different approaches to providing access to communication boards, including providing students with multiple communication boards each (Cases A and D), all sharing the same board (Case C), and having all communication boards placed on the whiteboard (Case B). These various approaches may reflect teachers’ considerations of classroom setups and student needs, factors known to influence AAC use in classrooms (Andzik et al., 2016, 2017; Leatherman & Wegner, 2022).

All cases had ceiling effects on items of the RAACS scale, hindering the tracking of changes over time on certain responsive strategies. Cases A and C, conducting the circle time gathered around a table, showed ceiling effects on Item 2: the teacher adjusts physically to the student. All cases had ceiling effects on two additional items, Item 4: the teacher clarifies their own communication, and Item 7: the teacher uses AAC. Several other studies have acknowledged ceiling effects on Items 2 and 4 (Broberg et al., 2012; Lindberger et al., 2024; Wandin et al., 2022), with efforts to address them in version 4 of the scale (Lindberger et al., 2024). Interestingly, studies on parents did not find ceiling effects on AAC use (Item 7 in RAACS; Broberg et al., 2012; Lindberger et al., 2024; Rensfeldt Flink et al., 2022), indicating that parents may not possess the same level of prior AAC skills as teachers working with students with ID.

In all observed classrooms, a consistently high staff/student ratio (ranging from 0.88 to 1.23) was evident, of which all staff members took part in the intervention. Previous research has shown that support staff can successfully use communication partner strategies (Chazin et al., 2021; Douglas, 2012) and that teacher and support staff collaboration can influence AAC implementation (Andzik et al., 2017; Biggs & Hacker, 2021; Leatherman & Wegner, 2022). As a result, the involvement of support staff in these classrooms may have influenced the teachers’ strategy use, especially given the substantial number of present adults.

Case B also consistently scored lower on responsive strategies than the other cases, again possibly due to the unique characteristic of having only speaking students. Research has shown that professionals employ responsive strategies to various degrees in interactions with different individuals (Popich & Alant, 1999; Shalev & Hetzroni, 2020; Wandin et al., 2022). However, studies further suggest that teachers are generally more responsive to verbal students (Popich & Alant, 1999; Shalev & Hetzroni, 2020), contradicting this initial speculation. Alternatively, these variations could result from individual differences in interaction patterns or teaching approaches. Likewise, the consistently lower augmented input frequency in Case C may be attributed to variations in interaction style, just as speech rate differs among speakers. The lower frequency in this case could also be the circumstance of having only one shared communication board, as described in the previous section.

### Limitations and future directions

Like any typical case study, this study has some limitations that influence the interpretation of its findings. The small sample size and few observational time points prevent generalization of the results to a broader population of school staff, necessitating caution in drawing conclusions. Nevertheless, the findings provide valuable insights into partner strategies and contextual factors in the cases examined. They inform future research and practice of similar communication partner interventions, demonstrating that teachers can utilize augmented input and responsive strategies as a universal classroom approach following intervention.

This study did not address the teachers’ spoken language, which could have provided additional insights into the application of augmented input. For instance, Senner and Baud (2017) utilized a percentage value to relate the frequency of augmented input to the number of spoken utterances. Future studies should also explore the communicative functions for which teachers use augmented input, informed by, for example, the work of Bunning et al. (2010). Additionally, it would be beneficial to investigate whether teachers’ communication and language were adapted to the communicative levels of their students.

As previously mentioned, ceiling effects on the RAACS scale hindered the tracking of changes over time. Additionally, this study's reliability assessments suggest that agreement on the scale could be improved. Conducting a forthcoming psychometric study in educational settings, following the design of prior research by Broberg et al. (2012) and Lindberger et al. (2024), would help improve the scale's applicability and agreement for use in school settings.

This study relied solely on observational data, without considering teacher perspectives on their use of communication partner strategies. It also did not assess the intervention application of other school staff, despite being a team-wide effort. This approach was taken because only teachers were consistently present across time points. Future studies should address these aspects and potentially assess the feasibility of such interventions in settings with lower staff/student ratios, as not all schools can offer the same level of support as those in the current study.

Finally, future research should also evaluate student communication. The focus on teacher behavior in this study was to ensure that teachers can apply the intervention before expecting it to influence student communication. Understanding teacher behavior also provides a solid foundation for future interventions focused on supporting teachers in facilitating student communication.

### Implications for practice

The findings suggest that teachers can use augmented input and responsive strategies as a universal approach in the classroom, and develop communication boards for this purpose, following a communication partner intervention. Notably, the three teachers selected by their principals to become ComPal instructors because of their high initial AAC skills showed the most consistent utilization. As the intervention aims to engage all school staff, this information is especially important for school administrators concerned about highly skilled staff not optimizing their time when partaking in such interventions.

The study findings further indicate that regularly nurturing augmented input and communication board access may be necessary. In educational settings, the intervention could be conducted periodically to sustain partner strategy use over time and introduce new staff to the concept while fostering team collaboration. Those who participate for the second time may find it easier to grasp and employ more challenging content, such as using augmented input in more spontaneous interactions.

Classroom contexts appear to influence the application of augmented input and responsive strategies. Only Cases A and C, seated at tables, demonstrated optimal physical positioning and continuous access to communication boards. Given the constraints of limited classroom space, it is important to explore alternative solutions to ensure optimal teacher positioning and communication board access.

It is noteworthy that only one student across all participating classrooms had access to an individualized communication system, despite multiple students being nonverbal. While teachers likely recognize their students’ communicative needs, the selection and access to personalized communication systems often fall outside the control of most school staff. Therefore, it is encouraging that this study suggests teachers can apply communication partner strategies as a universal approach in the classroom, potentially also benefiting future generations of students with communicative needs. However, ensuring access to individualized communication systems remains crucial, both in and beyond the classroom, to support the communication rights of each individual.

## Conclusions

The findings of this study indicate that a communication partner intervention with a universal classroom approach, the AKKtiv ComPal intervention, can be applied as intended by teachers for students with ID. All teachers increased their use of communication partner strategies postintervention, consistent with previous research. However, variability in the utilization of partner strategies across teachers, especially at follow-up, emphasizes the importance of ongoing support to sustain teachers’ strategy use. Differences in teacher application seem to be partly influenced by contextual factors such as classroom setups (e.g., having a table or not), student characteristics (e.g., being verbal or nonverbal), as well as teachers’ previous AAC skills. Furthermore, although previous research has underscored the benefits of the strategies included in the intervention, more studies are essential to directly assess the impact of the AKKtiv ComPal intervention on student communication outcomes.
